# Association of gut microbiome with risk of intracranial aneurysm: a mendelian randomization study

**DOI:** 10.1186/s12883-023-03288-2

**Published:** 2023-07-15

**Authors:** Chencheng Ma, Weiwei Zhang, Lei Mao, Guangjian Zhang, Yuqi Shen, Hanxiao Chang, Xiupeng Xu, Huiru Jin, Zheng Li, Hua Lu

**Affiliations:** 1 Department of Neurosurgery, The First Affiliated Hospital of Nanjing Medical University, Nanjing, Jiangsu Province China; 2 Department of Neurosurgery, Jiangsu Province Hospital, Nanjing, Jiangsu Province China; 3 Department of Ophthalmology, Third Medical Center of Chinese, PLA General Hospital, Beijing, China; 4 Department of Infectious Diseases, The First Affiliated Hospital of Nanjing Medical University, Nanjing, China

**Keywords:** Mendelian randomization, Intracranial aneurysm, Gut microbiome, cerebrovascular disease, Causality

## Abstract

**Objective:**

To investigate the potential causal link between genetic variants associated with gut microbiome and risk of intracranial aneurysm (IA) using two-sample mendelian randomization (MR).

**Methods:**

We performed two sets of MR analyses. At first, we selected the genome-wide statistical significant(*P* < 5 × 10^–8^) single nucleotide polymorphisms (SNPs) as instrumental variables (IVs). Then, we selected the locus-wide significant (*P* < 1 × 10^–5^) SNPs as IVs for the other set of analyses to obtain more comprehensive conclusions. Gut microbiome genetic association estimates were derived from a genome-wide association study (GWAS) of 18,473 individuals. Summary-level statistics for IA were obtained from 79,429 individuals, which included 7,495 cases and 71,934 controls.

**Results:**

On the basis of locus-wide significance level, inverse variance weighted(IVW) showed that *Clostridia* [(odds ratio (OR): 2.60; 95% confidence interval (CI): 1.00—6.72, *P* = 0.049)], *Adlercreutzia* (OR: 1.81; 95% CI: 1.10—2.99, *P* = 0.021) and *Victivallis* (OR: 1.38; 95% CI: 1.01—1.88, *P* = 0.044) were positively related with the risk of unruptured intracranial aneurysm(UIA); Weighted median results of MR showed *Oscillospira* (OR: 0.37; 95% CI: 0.17—0.84, *P* = 0.018) was negatively with the risk of UIA and *Sutterella* (OR: 1.84; 95% CI: 1.04—3.23, *P* = 0.035) was positively related with the risk of UIA; MR-Egger method analysis indicated that *Paraprevotella* (OR: 0.32; 95% CI: 0.13—0.80, *P* = 0.035) was negatively with the risk of UIA and *Rhodospirillaceae* (OR: 13.39; 95% CI: 1.44—124.47, *P* = 0.048) was positively related with the risk of UIA. The results suggest that *Streptococcus* (OR: 5.19; 95% CI: 1.25—21.56; *P* = 0.024) and *Peptostreptococcaceae* (OR: 4.92; 95% CI: 1.32—18.32; *P* = 0.018) may increase the risk of UIA according to genome-wide statistical significance thresholds.

**Conclusion:**

This MR analysis indicates that there exists a beneficial or detrimental causal effect of gut microbiota composition on IAs.

**Supplementary Information:**

The online version contains supplementary material available at 10.1186/s12883-023-03288-2.

## Introduction

Intracranial aneurysm (IA) is confined, pathological dilatations of the walls of intracranial arteries that are at risk of rupture. About 85% of spontaneous subarachnoid hemorrhage(SAH) is due to ruptured IA [1]. The incidence of IA was reported to be about 3.2% in a worldwide study with a mean age of 50 years [2]. Aneurysmal subarachnoid hemorrhage(aSAH) often has a poor prognosis(30% of death, 30% of independence and 30% of dependence), with patients often suffering from a disability or even death [3, 4]. Nonetheless, the etiology of IAs is as yet not completely perceived. If the causes of intracranial aneurysms could be prevented this would greatly reduce human suffering.

Recently, the relationship between IAs and gut microbiome has attracted a lot of attention. Gut flora has been found to assume a part in cardiovascular diseases such as atherosclerosis and hypertension [5, 6]. Hypertension and other related factors have long been reported to be significantly associated with intracranial aneurysms [7]. Therefore, we can speculate that intestinal dysbiosis may increase the risk of intracranial aneurysm through these high-risk factors for intracranial aneurysm. One study reported that eliminating intestinal flora with antibiotics significantly reduced the incidence of IAs in mice [8]. Another study found the abundance of certain intestinal flora was higher in patients with aSAH than in those with unruptured intracranial aneurysm(UIA) [9]. A recent study reported that after transplanting feces from UIA patients into mice, mice implanted with feces from UIA patients had a greatly increased risk of aneurysm and aneurysm rupture compared to feces from healthy humans [10]. Nevertheless, whether there exists a clear causal connection between IAs and intestinal microbiota is unclear.

Mendelian randomization (MR) is the use of genetic variation in non-experimental data to estimate the causal link between exposure and outcome, which can reduce the impact of behavioral, social, psychological, and other factors [11]. Using recently published summary data for gut microbiome and summary data for IA in genome-wide association study (GWAS), we aimed to analyze the causal connection between intestinal microbiota and IA through two-sample MR.

## Material and methods

### Genetic instruments and data sources

SNPs related with human gut microbiome were used in a GWAS. This GWAS included 18,340 individuals [11]. This is a large-scale multi-ethnic GWAS, mostly of European ancestry, containing 122,110 variant loci to explore the human genetic impact on gut microbiome composition.

SNPs associated with IA were extracted from a large GWAS [12]. This GWAS involved 71,934 controls and 7,495 cases. MR analysis was conducted using a summary data from this GWAS. This dataset is a GWAS of European ancestry individuals including UIA-only (*n* = 2,070) versus controls (*n* = 71,934).

To confirm the causal relationship between gut microbiome and IA risk, the best IVs were selected following the following steps. A threshold of significant association with the gut microbiome was set for the selection of SNPs as IVs as the first step. We screened for SNPs with genome-wide statistical significance (*P* < 5 × 10–8) as IVs. To achieve overall results, another set of locus-wide significant(*P* < 1 × 10–5) SNPs was used as IVs. Second, one of the principles of the MR method: There must be no linkage disequilibrium (LD) between the selected IVs, because the existence of strong LD may lead to biased results. During our MR analysis, we reduced the LD by clumping the selected SNPs (clumping distance = 10,000 kb, R2 < 0.001). Finally, during MR analysis, it is important to ensure that SNPs affect outcome and exposure with only one allele. According to this principle, SNPs of the palindrome structure will be removed.

### Standard protocol approvals, registrations, and patient consents

The MR analysis used summary GWAS data publicly available from GWASs. Due to the fact that each of the original GWASs had obtained ethical approval and participant consent, they were not required.

### The assumptions of MR

In MR, genetic variation is viewed as an IV, and the basic conditions for genetic variation to satisfy this IV are listed: There are no confounding factors associated with gut microbiome or IA that correlate with IVs; IVs are directly associated with gut microbiome; unless exposure is associated with the IVs, the IVs do not affect the outcome [12]. It is commonly used to evaluate the strength of the correlation between exposure and IVs using the *F* statistic, whose formula is (*R^2* (*n*-*k*-*1*))/(*k* (*1*-*R^2*)). The number of exposure samples in the GWAS study is *n*, the number of IVs is* k*, and the degree to which IVs explain exposure is *R^2*. It is usually considered a weak IV when the *F* statistic is less than 10, which may affect the results.

### Statistical analysis

A causal link between IAs and intestinal microbes was investigated using the inverse variance weighted (IVW) method, MR-Egger, weighted median, and weighted mode. In the IVW method, the intercept term is not considered. In this method, weights are based on the inverse of variance (quadratic of standard error). A comprehensive estimate of the impact of gut microbiota on IA incidence was obtained using this method. IVW results would be highly biased if these SNPs were pleiotropic, and this must be ensured. With MR-Egger, causal estimates are unaffected by breaches of the standard IV assumptions and violations of the standard IV assumptions can be detected [13]. Weighted medians combine information from different hereditary variations into one causal gauge, which is predictably accurate even with half of the null IVs [14].

An MR-Egger regression was conducted to determine whether the SNPs included had horizontal pleiotropy. In order to monitor the presence of IVs with horizontal polymorphisms, mendelian randomized pleiotropy residuals and outliers (MR-PRESSO) was used because it had better statistical power and accuracy than MR-Egger regression. MR-PRESSO was also used to correct horizontal polymorphisms. We performed leave-one-out analysis in order to determine if causal effect estimates were reliable in the presence of potentially strong impact SNPs. We performed a leave-one-out analysis to determine if there are potentially strong impact SNPs. Thus, we are able to test whether the causal effect estimates are reliable. A further examination of the heterogeneity among selected SNPs was conducted with Cochran's Q statistics. We used the R (version 4.1.2) packages MRPRESSO and TwoSampleMR to perform MR analyses.

## Results

### Instrumental variables selection

Firstly, we screened 461 (genome-wide statistical significance threshold, *P* < 5 × 10^–8^) and 10,417 (locus-wide significance level, *P* < 1 × 10^–5^) SNPs as IVs from a massive gut microbiome GWAS containing 211 taxa which consist of five biotypes of the genus, family, order, phylum, and class. After the removal of SNPs with LD and independent of IA, 12 (*P* < 5 × 10^–8^) and 1,291 (*P* < 1 × 10^–5^) SNPs remained as IVs. We collected important information of SNPs including beta, SE, *P*-value, effect allele, and other allele for further study.

### Locus-wide significance level with UIA

Weighted median results of MR showed *Oscillospira* [(odds ratio (OR): 0.37; 95% confidence interval (CI): 0.17—0.84, *P* = 0.018)] was negative with the occurrence of UIA and *Sutterella* (OR: 1.84; 95% CI: 1.04—3.23, *P* = 0.035) was positively related with the occurrence of UIA (Supplementary Table 1). The results of the MR-Egger method analysis indicated that *Paraprevotella* (OR: 0.32; 95% CI: 0.13—0.80, *P* = 0.035) was negative with the occurrence of UIA and *Rhodospirillaceae* (OR: 13.39; 95% CI: 1.44—124.47, *P* = 0.048) was positively related with the occurrence of UIA (Supplementary Table 1). The results of the IVW method analysis indicated that *Clostridia* (OR: 2.60; 95% CI: 1.00—6.72, *P* = 0.049), *Adlercreutzia* (OR: 1.81; 95% CI: 1.10—2.99, *P* = 0.021) and *Victivallis* (OR: 1.38; 95% CI: 1.01—1.88, *P* = 0.044) were positively related with the occurrence of UIA (Supplementary Table 1).

**Fig. 1:**
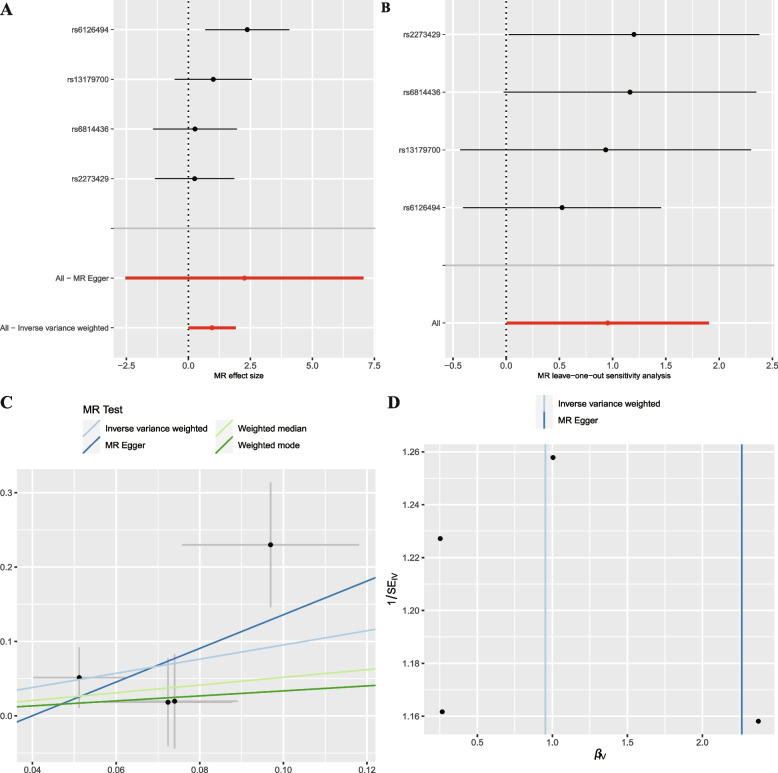
Forest plot (**A**), sensitivity analysis (**B**), scatter plot (**C**), and funnel plot (**D**) of the causal effect of *Clostridia* on IA risk

We used MR-Egger regression to evaluate the horizontal pleiotropy between IVs and outcomes, which indicated there existed horizontal pleiotropy between the instrument variables of *Paraprevotella* and outcomes (*P* = 0.029), and there was no evidence of horizontal pleiotropy between other IVs and outcomes (Supplementary Table 1). However, when further analyzed by MR-PRESSO, none of them were found to be horizontally pleiotropic. There existed no outliers in the MR-PRESSO analysis of *Clostridia* (*P* = 0.359), *Rhodospirillaceae* (*P* = 0.372), *Adlercreutzia* (*P* = 0.474), *Oscillospira* (*P* = 0.137), *Paraprevotella* (*P* = 0.172), *Sutterella* (*P* = 0.201), and *Victivallis* (*P* = 0.448). Details of the instrument variables are shown in Supplementary Table 2. All F-statistic values are greater than 10, which indicates the absence of weak IVs (Supplementary Table 1). Therefore, this study found that *Clostridia* (Fig. 1), *Rhodospirillaceae* (Supplementary Fig. 1), *Adlercreutzia* (Supplementary Fig. 2), *Sutterella* (Supplementary Fig. 3), *and Victivallis* (Supplementary Fig. 4) were occurrence factors for UIA and that *Oscillospira* (Supplementary Fig. 5) and *Paraprevotella* (Supplementary Fig. 6) played protective roles in the development of UIA.

### Genome-wide statistical significance threshold

When analyzing the association of intestinal microbiota overall with UIA, the results of MR Egger (OR: 0.92; 95% CI: 0.43—1.96, *P* = 0.837), weighted mode (OR: 1.13; 95% CI: 0.83—1.55;* P* = 0.458), weighted median (OR: 1.12; 95% CI: 0.83—1.52; *P* = 0.447) and IVW (OR: 1.19; 95% CI: 0.92—1.54; *P* = 0.187) showed that there exists no association between intestinal microbiota and UIA (Table 1 and Supplementary Fig. 7).

**Table 1:** MR results of causal links between gut microbiome and IA (*P* < 5 × 10–8)

Classification		Nsnp	Methods	Beta	SE	OR (95%CI)	*P* value	Horizontal pleiotropy			Heterogeneity		*F* statistic
								Egger intercept	SE	*P* value	Cochran’s Q	*P* value	
Unruptured IA													
Total		12	MR Egger	-0.08	0.39	0.92(0.43,1.96)	0.837	0.03	0.05	0.495	14.38	0.156	87.19
			Weighted median	0.12	0.15	1.12(0.83,1.52)	0.447						
			Inverse variance weighted	0.17	0.13	1.19(0.92,1.54)	0.187						
			Weighted mode	0.12	0.16	1.13(0.83,1.55)	0.458						
Class	*Melainabacteria*	1	Wald ratio	0.47	0.34	1.59(0.82,3.09)	0.169	–	–	–	–	–	–
Family	*Oxalobacteraceae*	1	Wald ratio	0.31	0.37	1.37(0.66,2.82)	0.400	–	–	–	–	–	–
	*Peptostreptococcaceae*	1	Wald ratio	1.59	0.67	4.92(1.32,18.32)	0.018	–	–	–	–	–	–
	*Streptococcaceae*	1	Wald ratio	-0.09	0.53	0.92(0.32,2.59)	0.870	–	–	–	–	–	–
	*unknownfamily (id:1,000,001,214)*	1	Wald ratio	-0.08	0.50	0.92(0.34,2.47)	0.870	–	–	–	–	–	–
Genus	*Eubacteriumcoprostanoligenesgroup*	1	Wald ratio	-0.72	0.73	0.49(0.12,2.03)	0.322	–	–	–	–	–	–
	*Eubacteriumnodatumgroup*	1	Wald ratio	0.13	0.25	1.14(0.70,1.87)	0.591	–	–	–	–	–	–
	*Ruminococcustorquesgroup*	1	Wald ratio	0.62	0.64	1.85(0.52,6.54)	0.339	–	–	–	–	–	–
	*Allisonella*	1	Wald ratio	-0.04	0.23	0.97(0.61,1.52)	0.878	–	–	–	–	–	–
	*Erysipelatoclostridium*	1	Wald ratio	-0.27	0.30	0.76(0.42,1.37)	0.361	–	–	–	–	–	–
	*Intestinibacter*	1	Wald ratio	-0.37	0.47	0.69(0.27,1.73)	0.428	–	–	–	–	–	–
	*Oxalobacter*	1	Wald ratio	-0.39	0.55	0.68(0.23,1.99)	0.476	–	–	–	–	–	–
	*RuminococcaceaeUCG013*	1	Wald ratio	0.32	0.37	1.37(0.67,2.81)	0.386	–	–	–	–	–	–
	*Streptococcus*	1	Wald ratio	1.65	0.73	5.19(1.25,21.56)	0.024	–	–	–	–	–	–
	*unknowngenus* (*id:1,000,001,215)*	1	Wald ratio	-0.08	0.50	0.92(0.34,2.47)	0.870	–	–	–	–	–	–
Order	*Gastranaerophilales*	1	Wald ratio	-0.08	0.50	0.92(0.34,2.47)	0.870	–	–	–	–	–	–

*Abbreviations*: *MR* Mendelian randomization, *IA* Intracranial aneurysm, *SNP* Single nucleotide polymorphism, *OR* Odds ratio, *CI* Confidence interval, *SE* Standard error

Specific information on IVs is provided in Table 2. MR-Egger regression indicated no horizontal pleiotropy in the analysis of the relationship between total gut microbiome and aneurysms (*P* = 0.495 for UIA). Besides, *F* statistics were more than 10, and Cochrane *Q* statistics showed no significant heterogeneity (*P* = 0.156 for UIA). The results of the intestinal microbiota classification suggested that *Streptococcus* (OR: 5.19; 95% CI: 1.25—21.56; *P* = 0.024) and *Peptostreptococcaceae* (OR: 4.92; 95% CI: 1.32—18.32; *P* = 0.018) may increase the occurrence of UIA (Table 1). The limited number of SNPs included prevented examination of horizontal pleiotropy and heterogeneity.

**Table 2:** SNPs used as instrumental variables from individual bacterial abundance, the whole gut microbiome and IA GWASs (*P* < 5 × 10–8)

Bacterial traits	SNP	Effect allele	Other allele	Gut microbiome		Unruptured IA	
				**Beta (SE)**	***P* value**	**Beta (SE)**	***P* value**
Total	rs10805326	G	A	0.08(0.01)	2.86E-08	-0.03(0.04)	0.477
	rs11110281	T	C	-0.14(0.02)	1.47E-09	0.01(0.07)	0.870
	rs12781711	C	T	-0.07(0.01)	2.33E-08	-0.11(0.05)	0.024
	rs17159861	C	T	0.10(0.02)	1.10E-08	-0.07(0.07)	0.322
	rs34297067	A	G	-0.19(0.03)	4.41E-08	-0.03(0.05)	0.592
	rs35866622	T	C	-0.06(0.01)	2.23E-08	-0.04(0.04)	0.338
	rs4428215	G	A	0.13(0.02)	4.76E-08	0.04(0.05)	0.400
	rs602075	A	G	0.17(0.03)	1.27E-08	-0.01(0.04)	0.878
	rs61841503	G	A	0.09(0.02)	1.19E-08	0.15(0.06)	0.018
	rs7221249	A	G	0.08(0.01)	4.01E-09	-0.03(0.04)	0.428
	rs736744	C	T	0.12(0.02)	2.41E-08	0.04(0.04)	0.385
	rs9864379	T	C	-0.16(0.03)	4.18E-08	-0.07(0.05)	0.169
*Melainabacteria*	rs9864379	T	C	-0.16(0.03)	4.76E-08	-0.07(0.05)	0.169
*Oxalobacteraceae*	rs4428215	G	A	0.13(0.02)	4.76E-08	0.04(0.05)	0.400
*Peptostreptococcaceae*	rs61841503	G	A	0.09(0.02)	1.19E-08	0.15(0.06)	0.018
*Streptococcaceae*	rs11110281	T	C	-0.13(0.02)	7.55E-09	0.01(0.07)	0.870
*unknownfamily (id:1,000,001,214)*	rs9864379	T	C	-0.16(0.03)	4.18E-08	-0.07(0.05)	0.169
*Eubacteriumcoprostanoligenesgroup*	rs17159861	C	T	0.10(0.02)	1.1E-08	-0.07(0.07)	0.322
*Eubacteriumnodatumgroup*	rs34297067	A	G	-0.19(0.03)	4.41E-08	-0.03(0.05)	0.592
*Ruminococcustorquesgroup*	rs35866622	T	C	-0.06(0.01)	2.23E-08	-0.04(0.04)	0.338
*Allisonella*	rs602075	A	G	0.17(0.03)	1.27E-08	-0.01(0.04)	0.878
*Erysipelatoclostridium*	rs7221249	A	G	0.08(0.01)	4.01E-09	-0.03(0.04)	0.428
*Intestinibacter*	rs10805326	G	A	0.08(0.01)	2.86E-08	-0.03(0.04)	0.477
*Oxalobacter*	rs736744	C	T	0.12(0.02)	2.41E-08	0.04(0.04)	0.385
*RuminococcaceaeUCG013*	rs12781711	C	T	-0.07(0.01)	2.33E-08	-0.11(0.05)	0.024
*Streptococcus*	rs11110281	T	C	-0.14(0.02)	1.47E-09	0.01(0.07)	0.870
*unknowngenus* (*id:1,000,001,215)*	rs9864379	T	C	-0.16(0.03)	4.18E-08	-0.07(0.05)	0.169
*Gastranaerophilales*	rs9864379	T	C	-0.16(0.03)	4.18E-08	-0.07(0.05)	0.169

*Abbreviations*: *MR* Mendelian randomization, *IA* Intracranial aneurysm, *SNP* Single nucleotide polymorphism, *SE* Standard error

## Discussion

Our MR analysis gives proof to prove that *Streptococcus*, *Adlercreutzia*, *Clostridia*, *Rhodospirillaceae*, *Sutterella*, *Victivallis* and *Peptostreptococcaceae* increase the occurrence of IA, *Oscillospira* and *Paraprevotella* are protective factors for IA. However, only a few IVs reached genome-wide statistically significant levels, making the accuracy of the results of *Streptococcus* and *Peptostreptococcaceae* potentially subject to some bias.

It has been realized by more and more neurosurgeons that environmental factors act a more significant role than inherited factors in the pathophysiology of IAs [15, 16]. Pyysalo et al. have reported the association of IAs with oral bacteria, suggesting a link between aneurysms and bacteria [17]. The gut microbiota may influence IA formation and rupture by modulating local inflammation and affecting blood pressure [18]. *Streptococcus* may increase the release of inflammatory factors such as IL-1β, IFN-γ, and IL-6 [19]. One study reported that *Streptococcus* is usually in higher abundance in hypertensive patients [20]. *Peptostreptococcaceae* promote the progression of atherosclerosis, which may promote aneurysm formation [21]. *Prevotella* was found in high levels in patients with rheumatoid arthritis and has been found to be associated with chronic inflammation [22, 23]. These reports are consistent with our findings. Nevertheless, specific ways of the effects of these intestinal florae on inflammation and aneurysms remain to be further investigated.

Although some studies have shown the presence of intestinal flora disorders in patients with IAs, this may only be a clinical symptom of IAs and there appears to be no causal association between IAs and intestinal flora disorders. On the one hand, the components of the intestinal microbiota might differ due to inconsistent sex ratios and ethnicity across studies. On the other hand, although IA patients were found to have dysbiosis of the intestinal microbiota, there is no agreement as to which strains play a key role. These unresolved issues have prevented inferring a causal association between the intestinal microbiota and the occurrence and rupture of IAs. In the treatment of intracranial aneurysms, it may be possible in the future to reduce the risk of occurrence and rupture of intracranial aneurysms by targeting certain bacteria for eradication.

To our knowledge, the present study is the first MR analysis of the relationship between IAs and intestinal microflora. The fundamental benefit of this MR analysis is that estimates of the causal effect of MR are not distorted by confounding factors and reverse causal associations found in traditional epidemiological studies. Therefore, compared to observational studies, it may be more persuasive. Yet, several limitations remain. First, in the two-sample MR analysis, we could not confirm whether overlapping participants participated in the exposures and outcomes GWAS. We use *F* statistics to minimize the bias of overlapping participants. Second, due to its biological plausibility and multistage statistical process, it may be too conservative and may omit potential strains that are causally associated with IA when applying rigorous multiple test correction. Therefore, we did not consider multiple tests. Third, due to most participants in GWAS being of European origin, it may be not applicable to other groups of people. Fourth, the original study lacked detailed demographic information, and further subgroup analysis was not possible. Finally, some studies have reported a progressive increase in the age of onset of subarachnoid hemorrhage [24]. Although it is true that studies have shown that the structure and species of the gut microbiota change with age, there may be some bias due to the absence of a specific age classification for the exposure factors selected for our study [25].

In summary, this MR research identified a causal impact of intestinal flora on IAs. Several intestinal microbiomes identified in this study that are associated with the occurrence and rupture of IAs may have the prospect of preventing IAs.

## Supplementary Information

**Additional file 1:** **SupplementaryFigure 1**. Forest plot (A),sensitivity analysis (B), scatter plot (C), and funnel plot (D) of the causaleffect of *Rhodospirillaceae *on UIA risk.

**Additional file 2:** **SupplementaryFigure 2**. Forest plot (A),sensitivity analysis (B), scatter plot (C), and funnel plot (D) of the causaleffect of *Adlercreutzia *on UIA risk.

**Additional file 3:** **SupplementaryFigure 3**. Forest plot (A),sensitivity analysis (B), scatter plot (C), and funnel plot (D) of the causaleffect of *Sutterella *on UIA risk.

**Additional file 4:** **SupplementaryFigure 4**. Forest plot (A),sensitivity analysis (B), scatter plot (C), and funnel plot (D) of the causaleffect of *Victivallis *on UIA risk.

**Additional file 5:** **SupplementaryFigure 5**. Forest plot (A),sensitivity analysis (B), scatter plot (C), and funnel plot (D) of the causaleffect of *Oscillospira *on UIA risk.

**Additional file 6:** **SupplementaryFigure 6**. Forest plot (A),sensitivity analysis (B), scatter plot (C), and funnel plot (D) of the causaleffect of *Paraprevotella *on UIA risk.

**Additional file 7:** **SupplementaryFigure 7**. Forest plot (A),sensitivity analysis (B), scatter plot (C), and funnel plot (D) of the causaleffect of the whole gut microbiome on UIA risk.

**Additional file 8:** **Supplementary Table 1.** MR results of causallinks between gut microbiome and UIA risk (*P* < 1 × 10^-5^). **Supplementary Table 2.** SNPs used asinstrumental variables from gut microbiome and UIA GWASs (*P* < 1 × 10^-5^).

## Data Availability

The data used in the article is presented in the article/supplementary material. Please contact the corresponding author for further information.
