# Supplementary material for: Association of gut microbiome with risk of intracranial aneurysm: a mendelian randomization study
Source: BMC Neurol. 2023 Jul 15;23:269. doi: 10.1186/s12883-023-03288-2 (PMC10349504; doi:10.1186/s12883-023-03288-2)
Supplement: Supplementary file 1 — Additional file 1: SupplementaryFigure 1. Forest plot (A),sensitivity analysis (B), scatter plot (C), and funnel plot (D) of the causaleffect of Rhodospirillaceae on UIA risk. [file 12883_2023_3288_MOESM1_ESM.pdf]

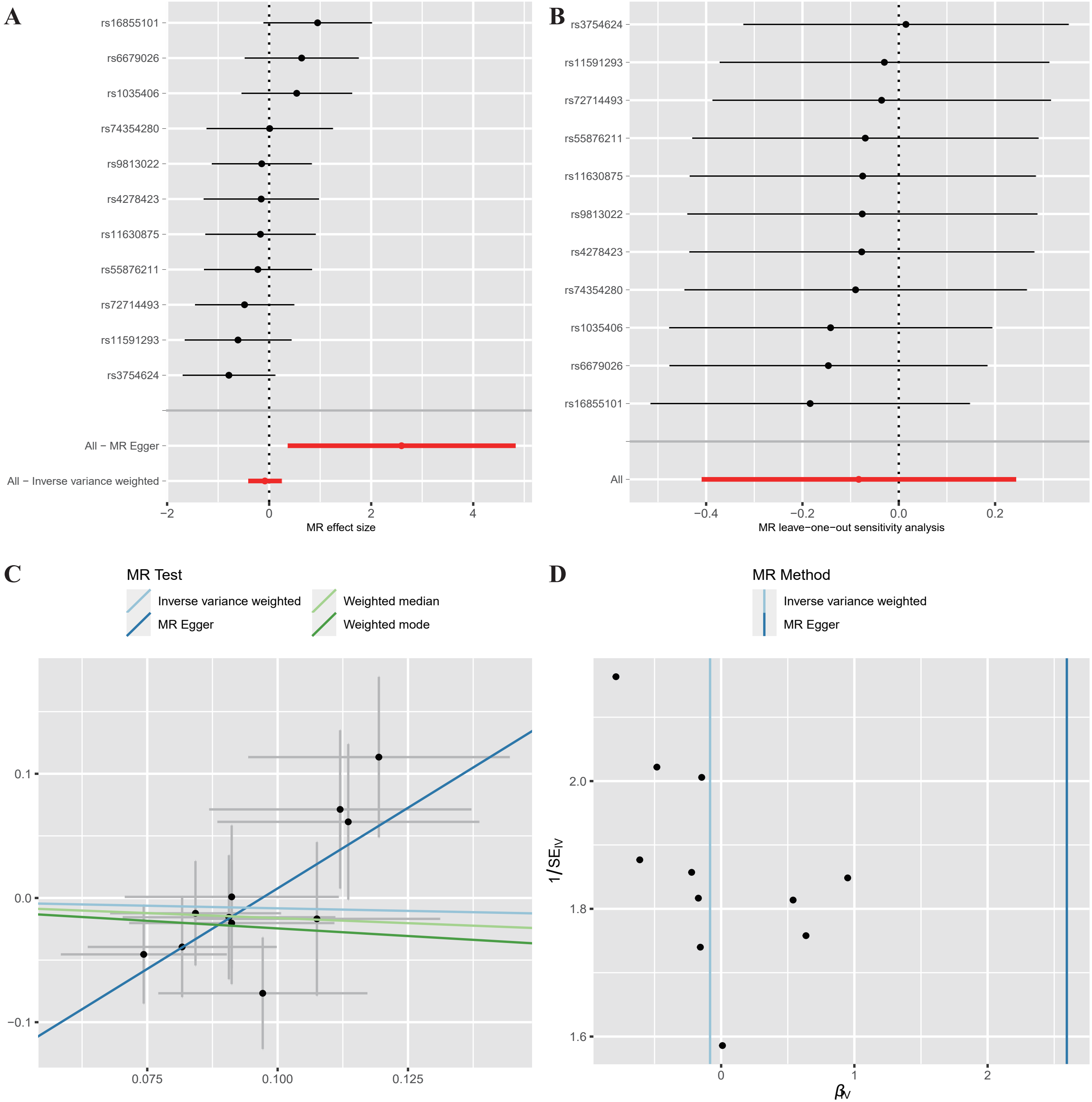

Supplementary Figure 1. Forest plot (A), sensitivity analysis (B), scatter plot (C), and funnel plot (D) of the causal effect of *Rhodospirillaceae* on UIA risk.
