# Supplementary material for: Association of gut microbiome with risk of intracranial aneurysm: a mendelian randomization study
Source: BMC Neurol. 2023 Jul 15;23:269. doi: 10.1186/s12883-023-03288-2 (PMC10349504; doi:10.1186/s12883-023-03288-2)
Supplement: Supplementary file 5 — Additional file 5: SupplementaryFigure 5. Forest plot (A),sensitivity analysis (B), scatter plot (C), and funnel plot (D) of the causaleffect of Oscillospira on UIA risk. [file 12883_2023_3288_MOESM5_ESM.pdf]

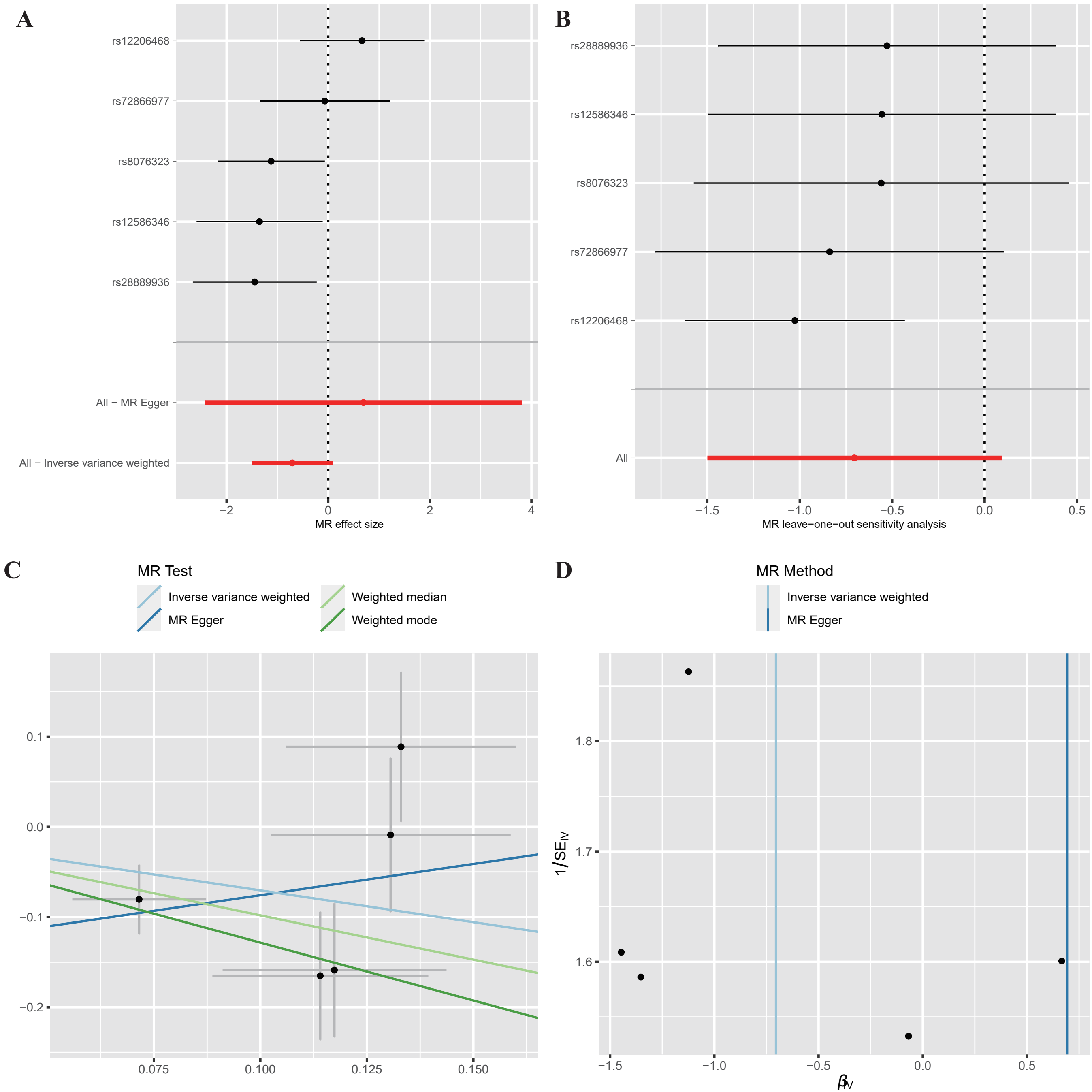

Supplementary Figure 5. Forest plot (A), sensitivity analysis (B), scatter plot (C), and funnel plot (D) of the causal effect of *Oscillospira* on UIA risk.
