# Supplementary material for: Association of gut microbiome with risk of intracranial aneurysm: a mendelian randomization study
Source: BMC Neurol. 2023 Jul 15;23:269. doi: 10.1186/s12883-023-03288-2 (PMC10349504; doi:10.1186/s12883-023-03288-2)
Supplement: Supplementary file 8 — Additional file 8: Supplementary Table 1. MR results of causallinks between gut microbiome and UIA risk (P < 1 × 10^-5^). Supplementary Table 2. SNPs used asinstrumental variables from gut microbiome and UIA GWASs (P < 1 × 10^-5^). [file 12883_2023_3288_MOESM8_ESM.docx]

Supplementary Table1.MR results of causal links between gut microbiome and UIA risk (P < 1 × 10^-5^).

| Classification |  | Nsnp | Methods | Beta | SE | OR (95%CI) | *P*  value | Horizontal pleiotropy | | |  | Heterogeneity | | *F* statistic |
| --- | --- | --- | --- | --- | --- | --- | --- | --- | --- | --- | --- | --- | --- | --- |
|  |  |  |  |  |  |  |  | Egger intercept | SE | *P*  value |  | Cochran’s Q | *P*  value |  |
| class | *Clostridia* | 4 | MR Egger | 2.26 | 2.45 | 9.60,0.08,1164.08 | 0.453 | -0.09 | 0.16 | 0.638 |  | 3.58 | 0.170 | 22.84 |
|  |  |  | Weighted median | 0.52 | 0.52 | 1.68,0.60,4.69 | 0.323 |  |  |  |  |  |  |  |
|  |  |  | Inverse variance weighted | 0.95 | 0.49 | 2.60,1.00,6.72 | 0.049 |  |  |  |  |  |  |  |
|  |  |  | Weighted mode | 0.34 | 0.64 | 1.40,0.40,4.88 | 0.635 |  |  |  |  |  |  |  |
| family | *Rhodospirillaceae* | 11 | MR Egger | 2.59 | 1.14 | 13.39,1.44,124.47 | 0.048 | -0.25 | 0.11 | 0.041 |  | 4.98 | 0.836 | 44.14 |
|  |  |  | Weighted median | -0.16 | 0.23 | 0.85,0.54,1.33 | 0.478 |  |  |  |  |  |  |  |
|  |  |  | Inverse variance weighted | -0.08 | 0.17 | 0.92,0.66,1.28 | 0.617 |  |  |  |  |  |  |  |
|  |  |  | Weighted mode | -0.24 | 0.37 | 0.78,0.38,1.63 | 0.528 |  |  |  |  |  |  |  |
| genus | *Adlercreutzia* | 4 | MR Egger | 1.47 | 0.93 | 4.36,0.70,27.23 | 0.256 | -0.09 | 0.09 | 0.431 |  | 1.84 | 0.399 | 50.60 |
|  |  |  | Weighted median | 0.56 | 0.31 | 1.76,0.95,3.24 | 0.071 |  |  |  |  |  |  |  |
|  |  |  | Inverse variance weighted | 0.59 | 0.26 | 1.81,1.10,2.99 | 0.021 |  |  |  |  |  |  |  |
|  |  |  | Weighted mode | 0.62 | 0.48 | 1.85,0.73,4.70 | 0.285 |  |  |  |  |  |  |  |
|  | *Oscillospira* | 5 | MR Egger | 0.69 | 1.59 | 2.00,0.09,45.05 | 0.692 | -0.15 | 0.16 | 0.430 |  | 6.94 | 0.074 | 41.41 |
|  |  |  | Weighted median | -0.98 | 0.41 | 0.37,0.17,0.84 | 0.018 |  |  |  |  |  |  |  |
|  |  |  | Inverse variance weighted | -0.70 | 0.41 | 0.49,0.22,1.09 | 0.082 |  |  |  |  |  |  |  |
|  |  |  | Weighted mode | -1.28 | 0.58 | 0.28,0.09,0.86 | 0.091 |  |  |  |  |  |  |  |
|  | *Paraprevotella* | 12 | MR Egger | -1.15 | 0.47 | 0.32,0.13,0.80 | 0.035 | 0.13 | 0.05 | 0.029 |  | 8.66 | 0.565 | 66.69 |
|  |  |  | Weighted median | -0.01 | 0.20 | 0.99,0.66,1.46 | 0.943 |  |  |  |  |  |  |  |
|  |  |  | Inverse variance weighted | 0.01 | 0.16 | 1.01,0.74,1.37 | 0.970 |  |  |  |  |  |  |  |
|  |  |  | Weighted mode | -0.39 | 0.38 | 0.68,0.32,1.43 | 0.327 |  |  |  |  |  |  |  |
|  | *Sutterella* | 11 | MR Egger | -0.36 | 1.03 | 0.70,0.09,5.24 | 0.734 | 0.05 | 0.07 | 0.477 |  | 13.06 | 0.160 | 31.09 |
|  |  |  | Weighted median | 0.61 | 0.29 | 1.84,1.04,3.23 | 0.035 |  |  |  |  |  |  |  |
|  |  |  | Inverse variance weighted | 0.38 | 0.24 | 1.46,0.92,2.32 | 0.105 |  |  |  |  |  |  |  |
|  |  |  | Weighted mode | 0.96 | 0.45 | 2.62,1.09,6.30 | 0.057 |  |  |  |  |  |  |  |
|  | *Victivallis* | 5 | MR Egger | -0.08 | 1.89 | 0.92,0.02,37.36 | 0.967 | 0.05 | 0.25 | 0.843 |  | 3.90 | 0.273 | 126.56 |
|  |  |  | Weighted median | 0.37 | 0.21 | 1.44,0.95,2.2 | 0.087 |  |  |  |  |  |  |  |
|  |  |  | Inverse variance weighted | 0.32 | 0.16 | 1.38,1.01,1.88 | 0.044 |  |  |  |  |  |  |  |
|  |  |  | Weighted mode | 0.58 | 0.32 | 1.78,0.96,3.32 | 0.143 |  |  |  |  |  |  |  |

Abbreviations: MR=mendelian randomization; UIA=unruptured intracranial aneurysm; SNP=single nucleotide polymorphism; SE=standard error.

Supplementary Table2.SNPs used as instrumental variables from gut microbiome and UIA GWASs (P < 1 × 10^-5^).

| **Bacterial traits** | **SNP** | **Effect allele** | **Other allele** | **Gut microbiome** | | | **UIA** | | |
| --- | --- | --- | --- | --- | --- | --- | --- | --- | --- |
|  |  |  |  | **Beta** | **SE** | ***P* value** | **Beta** | **SE** | ***P* value** |
| *Clostridia* | rs13179700 | T | C | 0.05 | 0.01 | 2.98E-06 | 0.05 | 0.04 | 0.206 |
|  | rs2273429 | A | G | -0.07 | 0.02 | 2.26E-06 | -0.02 | 0.06 | 0.756 |
|  | rs6126494 | G | T | -0.10 | 0.02 | 4.67E-06 | -0.23 | 0.08 | 0.006 |
|  | rs6814436 | T | C | 0.07 | 0.02 | 9.27E-07 | 0.02 | 0.06 | 0.757 |
| *Rhodospirillaceae* | rs1035406 | G | A | -0.11 | 0.03 | 6.01E-06 | -0.06 | 0.06 | 0.327 |
|  | rs11591293 | G | T | 0.07 | 0.02 | 2.84E-06 | -0.05 | 0.04 | 0.251 |
|  | rs11630875 | T | C | 0.09 | 0.02 | 8.38E-06 | -0.02 | 0.05 | 0.756 |
|  | rs16855101 | C | T | 0.12 | 0.03 | 1.87E-06 | 0.11 | 0.06 | 0.079 |
|  | rs3754624 | C | T | 0.10 | 0.02 | 1.20E-06 | -0.08 | 0.04 | 0.087 |
|  | rs4278423 | T | C | 0.11 | 0.02 | 5.08E-06 | -0.02 | 0.06 | 0.786 |
|  | rs55876211 | C | T | -0.09 | 0.02 | 3.43E-06 | 0.02 | 0.05 | 0.680 |
|  | rs6679026 | T | C | 0.11 | 0.03 | 8.20E-06 | 0.07 | 0.06 | 0.264 |
|  | rs72714493 | A | G | 0.08 | 0.02 | 6.24E-06 | -0.04 | 0.04 | 0.330 |
|  | rs74354280 | C | T | -0.09 | 0.02 | 8.58E-06 | -0.01 | 0.06 | 0.988 |
|  | rs9813022 | A | G | -0.08 | 0.02 | 2.61E-07 | 0.01 | 0.04 | 0.770 |
| *Adlercreutzia* | rs13231526 | C | A | 0.14 | 0.03 | 4.31E-06 | 0.16 | 0.07 | 0.017 |
|  | rs34181676 | G | T | -0.13 | 0.03 | 1.74E-06 | -0.05 | 0.07 | 0.518 |
|  | rs7680684 | C | T | -0.08 | 0.02 | 7.95E-07 | -0.06 | 0.04 | 0.151 |
|  | rs9915817 | T | C | 0.07 | 0.02 | 8.55E-06 | -0.01 | 0.04 | 0.943 |
| *Oscillospira* | rs12206468 | G | A | -0.13 | 0.03 | 8.17E-07 | -0.09 | 0.08 | 0.286 |
|  | rs12586346 | A | G | 0.12 | 0.03 | 7.60E-06 | -0.16 | 0.07 | 0.032 |
|  | rs28889936 | A | C | 0.11 | 0.03 | 6.46E-06 | -0.17 | 0.07 | 0.020 |
|  | rs72866977 | A | C | -0.13 | 0.03 | 3.56E-06 | 0.01 | 0.09 | 0.917 |
|  | rs8076323 | A | G | 0.07 | 0.02 | 4.88E-06 | -0.08 | 0.04 | 0.036 |
| *Paraprevotella* | rs13023298 | A | G | 0.10 | 0.02 | 9.08E-06 | 0.05 | 0.05 | 0.344 |
|  | rs17109926 | A | G | -0.10 | 0.02 | 4.83E-06 | 0.06 | 0.05 | 0.208 |
|  | rs17785622 | A | G | 0.25 | 0.05 | 2.23E-06 | -0.16 | 0.09 | 0.070 |
|  | rs2081023 | A | G | -0.12 | 0.02 | 2.19E-07 | -0.05 | 0.06 | 0.412 |
|  | rs3008582 | T | C | 0.11 | 0.02 | 3.28E-06 | -0.04 | 0.05 | 0.469 |
|  | rs3801748 | G | A | 0.08 | 0.02 | 5.59E-06 | 0.04 | 0.04 | 0.275 |
|  | rs4756632 | G | T | -0.14 | 0.03 | 1.65E-06 | -0.03 | 0.07 | 0.675 |
|  | rs4767113 | C | T | 0.09 | 0.02 | 1.58E-06 | -0.01 | 0.04 | 0.841 |
|  | rs58117850 | C | A | -0.15 | 0.03 | 3.68E-06 | 0.07 | 0.08 | 0.375 |
|  | rs77693755 | C | T | -0.14 | 0.03 | 4.26E-06 | 0.05 | 0.08 | 0.526 |
|  | rs9602779 | A | C | -0.11 | 0.02 | 1.27E-06 | -0.01 | 0.04 | 0.845 |
|  | rs9900242 | A | G | -0.09 | 0.02 | 1.13E-06 | -0.08 | 0.03 | 0.018 |
| *Sutterella* | rs13173038 | A | G | -0.07 | 0.02 | 2.18E-06 | -0.06 | 0.04 | 0.176 |
|  | rs143438747 | T | C | -0.15 | 0.03 | 2.02E-06 | -0.03 | 0.09 | 0.726 |
|  | rs2050185 | G | A | 0.06 | 0.01 | 7.95E-06 | 0.05 | 0.04 | 0.211 |
|  | rs2321387 | G | A | -0.06 | 0.01 | 1.92E-06 | 0.03 | 0.04 | 0.491 |
|  | rs2613606 | C | T | -0.06 | 0.01 | 7.26E-06 | -0.06 | 0.04 | 0.126 |
|  | rs35716880 | T | C | -0.11 | 0.02 | 9.41E-06 | 0.02 | 0.07 | 0.788 |
|  | rs607327 | C | T | 0.06 | 0.01 | 7.42E-06 | 0.07 | 0.04 | 0.106 |
|  | rs62501473 | G | A | 0.07 | 0.01 | 3.38E-06 | 0.08 | 0.05 | 0.121 |
|  | rs7499539 | A | G | 0.06 | 0.01 | 2.43E-06 | -0.01 | 0.04 | 0.930 |
|  | rs7638039 | T | C | 0.06 | 0.01 | 7.22E-06 | -0.09 | 0.05 | 0.058 |
|  | rs9350083 | T | G | -0.06 | 0.01 | 9.50E-06 | -0.06 | 0.04 | 0.116 |
| *Victivallis* | rs11899949 | G | A | 0.13 | 0.03 | 2.28E-06 | -0.01 | 0.04 | 0.925 |
|  | rs173120 | T | C | 0.13 | 0.03 | 3.99E-06 | 0.09 | 0.05 | 0.112 |
|  | rs1882775 | A | G | -0.14 | 0.03 | 9.81E-06 | 0.01 | 0.06 | 0.837 |
|  | rs56349194 | A | G | -0.16 | 0.03 | 4.95E-07 | -0.07 | 0.05 | 0.168 |
|  | rs911666 | T | C | -0.12 | 0.03 | 6.60E-06 | -0.08 | 0.04 | 0.060 |

Abbreviations: UIA=unruptured intracranial aneurysm; SNP=single nucleotide polymorphism; SE=standard error.
